# Efficacy and safety of insulin in type 2 diabetes: meta-analysis of randomised controlled trials

**DOI:** 10.1186/s12902-016-0120-z

**Published:** 2016-07-08

**Authors:** Sylvie Erpeldinger, Michaela B. Rehman, Christophe Berkhout, Christophe Pigache, Yves Zerbib, Francis Regnault, Emilie Guérin, Irène Supper, Catherine Cornu, Behrouz Kassaï, François Gueyffier, Rémy Boussageon

**Affiliations:** University college of General Medicine, University Claude Bernard Lyon 1, Lyon, France; Department of Cardiology, CHU de Poitiers, 86000 Poitiers, France; Department of General Medicine, University Lille-Nord de France, Lille 2, Lille, France; SCF SHS/S2HEP (EA 4148), University Claude Bernard Lyon 1, Lyon, France; UMR 5558, CNRS, Université Claude Bernard Lyon 1, Lyon, France; Clinical Investigation Centre, INSERM CIC1407, Lyon, France; Clinical Pharmacology and Clinical Trials Department, Hospices Civils de Lyon, Lyon, France; Department of General Medicine, Université de Poitiers, Poitiers, France

**Keywords:** Meta-analysis, Type 2 Diabetes Mellitus, Hypoglycaemic drugs, Insulin, Mortality, Morbidity, Randomised Controlled Trials

## Abstract

**Background:**

It is essential to anticipate and limit the social, economic and sanitary cost of type 2 diabetes (T2D), which is in constant progression worldwide.

When blood glucose targets are not achieved with diet and lifestyle intervention, insulin is recommended whether or not the patient is already taking hypoglycaemic drugs. However, the benefit/risk balance of insulin remains controversial. Our aim was to determine the efficacy and safety of insulin vs. hypoglycaemic drugs or diet/placebo on clinically relevant endpoints.

**Methods:**

A systematic literature review (Pubmed, Embase, Cochrane Library) including all randomised clinical trials (RCT) analysing insulin vs. hypoglycaemic drugs or diet/placebo, published between 1950 and 2013, was performed. We included all RCTs reporting effects on all-cause mortality, cardiovascular mortality, death by cancer, cardiovascular morbidity, microvascular complications and hypoglycaemia in adults ≥ 18 years with T2D. Two authors independently assessed trial eligibility and extracted the data. Internal validity of studies was analyzed according to the Cochrane Risk of Bias tool. Risk ratios (RR) with 95 % confidence intervals (95 % CI) were calculated, using the fixed effect model in first approach. The I^2^ statistic assessed heterogeneity. In case of statistical heterogeneity, subgroup and sensitivity analyses then a random effect model were performed. The alpha threshold was 0.05. Primary outcomes were all-cause mortality and cardiovascular mortality. Secondary outcomes were non-fatal cardiovascular events, hypoglycaemic events, death from cancer, and macro- or microvascular complications.

**Results:**

Twenty RCTs were included out of the 1632 initially identified studies. 18 599 patients were analysed: Insulin had no effect vs. hypoglycaemic drugs on all-cause mortality RR = 0.99 (95 % CI =0.92–1.06) and cardiovascular mortality RR = 0.99 (95 % CI =0.90–1.09), nor vs. diet/placebo RR = 0.92 (95 % CI = 0.80–1.07) and RR = 0.95 (95 % CI 0.77–1.18) respectively. No effect was found on secondary outcomes either. However, severe hypoglycaemia was more frequent with insulin compared to hypoglycaemic drugs RR = 1.70 (95 % CI = 1.51–1.91).

**Conclusions:**

There is no significant evidence of long term efficacy of insulin on any clinical outcome in T2D. However, there is a trend to clinically harmful adverse effects such as hypoglycaemia and weight gain. The only benefit could be limited to reducing short term hyperglycemia. This needs to be confirmed with further studies.

**Electronic supplementary material:**

The online version of this article (doi:10.1186/s12902-016-0120-z) contains supplementary material, which is available to authorized users.

## Background

In 2030, according to the World Health Organization [1], 366 million people worldwide will live with type 2 diabetes (T2D). This increase is linked to aging of the population, the rise of obesity, the change in diagnostic criteria of diabetes and more extensive screening [2]. Compared with the non-diabetic population of the same age, all-cause mortality: (hazard ratio: 1.80 (95 % CI: 1.71 to 1.90) and cardiovascular mortality: 2.32 (95 % CI: 2.11–2.56) are increased in T2D [2].

Insulin is a natural vital treatment in type 1 diabetes, because of the total absence of insulin secretion. In T2D the lack of insulin is relative and endogenous insulin levels are very high. [3, 4] In T2D, insulin is often prescribed when blood glucose targets are not met despite maximal dosages and combinations of oral hypoglycaemic drugs.

The American Diabetes Association (ADA) and the European Association for the Study of Diabetes (EASD) published a consensus statement recommending insulin as a well-validated tier 1 option for the metabolic management of hyperglycaemia in T2D [5].

ADA/EASD [5] and the NICE [6] guidelines recommend metformin as the first line oral hypoglycaemic agent. However, although metformin is still considered as the first line drug, a recent meta-analysis has shown a controversial benefit/risk balance for metformin [7].

Another recent meta-analysis [8] demonstrated no efficacy of sulfonylureas on cardiovascular morbidity or mortality in T2D. Furthermore, studies analysing the efficacy of metformin or sulfonylureas on clinical endpoints are scarce.

For these reasons, our objective is to analyse the efficacy of insulin on clinically relevant endpoints in T2D.

To answer this question we performed this meta-analysis of randomized clinical trials (RCT) analysing the short, medium and long-terms effects of insulin on clinical outcomes in T2D. Clinical outcomes were defined as mortality, morbidity and main adverse effects (such as total and severe hypoglycaemic events).

## Methods

Our methodology adheres to the PRISMA guidelines (see PRISMA checklist: Additional file 1)

### Data sources

Clinical trials were identified searching: Pubmed, Embase and Cochrane Library. We included all trials, with no language resctriction, published from January 1950 to April 2013. Keywords used were: “type 2 diabetes”, “diabetes mellitus”; “mortality”; “sudden death”; “sudden death”, “cardiac”; “macrovascular”; “cardiovascular or coronary disease”; “stroke”; “peripheral vascular disease”; “microvascular”; “retinopathy”; “neuropathy”; “diabetic nephropathy”; “kidney disease”; “hypoglycaemia”; “hypoglycaemic agents”; “insulin”; “insulin, lente”; “insulin aspart”; “insulin lispro”; “short-acting insulin”, “long-acting”; “insulin isophane”; “insulin ultralente”. We restricted our search to randomized clinical trials (RCTs), systematic reviews and meta-analyses of RCTs. We manually searched the reference lists of systematic reviews to check they had all already been identified in our study.

Search strategy is illustrated in the Additional file 2.

### Study selection

We included RCTs comparing insulin regimens vs. a hypoglycaemic drug or placebo/diet in T2D patients aged 18 to 80 years. By excluding patients over the age of 80, we excluded patients in which moderate hyperglcaemia is sometimes accepted, to reduce the risk of hypoglycaemia. Insulin was either administered alone or in combination with another hypoglycaemic agent. For example: Insulin vs. hypoglycaemic dug, (Insulin + hypoglycaemic drug) vs. (hypoglycaemic drug), Insulin vs. placebo, Insulin vs. diet.

Two investigators independently assessed eligibility (FR and EG). In case of discrepancy, a third observer adjudicated the eligibility (FG or SE). The extraction forms and the risk of bias assessments are attached as Additional file 2.

### Quality assessment

Two authors (FR and EG) independently assessed trial quality. Internal validity was analyzed with the Cochrane Risk of Bias tool [9]. These articles were then rated according to methodological quality: good, moderate or low.

### Outcomes

Primary outcomes were all-cause mortality and cardiovascular mortality. Secondary outcomes were non-fatal cardiovascular events, (such as myocardial infarction and stroke), hypoglycaemic events (total and severe), death from cancer and macro- or microvascular complications (such as blindness, or retinopathy). Hypoglycaemia requiring the intervention of a third party was considered as severe.

Two reviewers (FR and EG) independently extracted the data for all the outcomes of interest.

### Principal summary measures and statistical analysis

Analyses were done using Revman software version 5 (www.cc-ims.net/revman).

For all studies we calculated risk ratios (RR) with 95 % confidence intervals, (95 % CI), using the fixed effect model in first approach. Heterogeneity was investigated with the I^2^ statistic. It measures the proportion of overall variation attributable to between study heterogeneity. I^2^ values of 25 %, > 50 % and > 75 % refers respectively to a low, substantial and considerable degree of heterogeneity. In case of statistical heterogeneity, we tried to explain this with subgroup and sensitivity analyses then with a random effect model. Statistical significance was defined with an alpha threshold at 0.05.

## Results

**Fig. 1:**
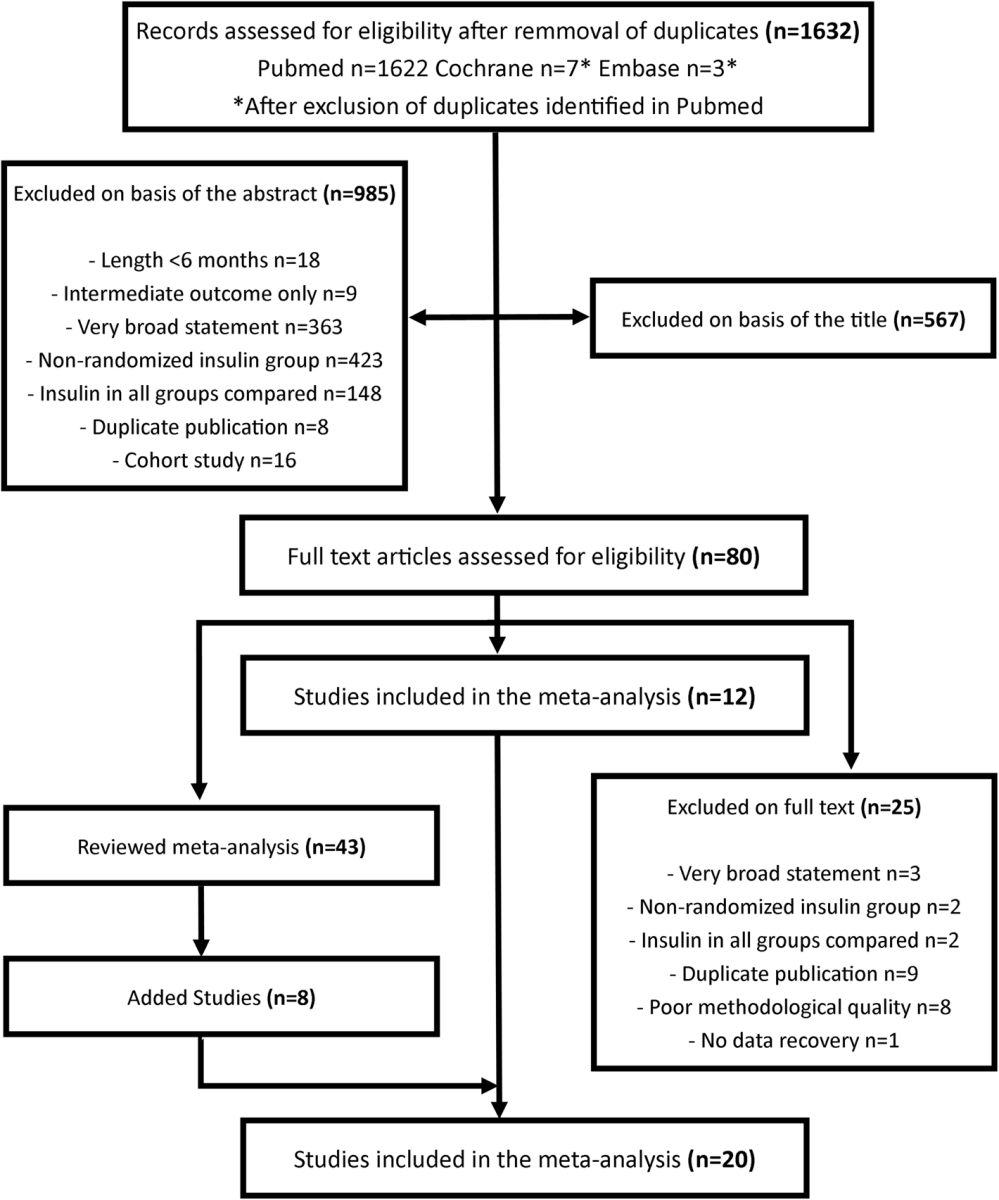
Study Flow Chart

Figure 1 shows the selection of studies. The list of the trials that were excluded after reviewing the abstract is available on request (under reasons for exclusion). 20 trials were included in the final analysis, 8 were identified as citations from other meta-analyses. The 20 trials compared insulin regimens vs. hypoglycaemic drugs (Table 1). Four of the 20 studies also compared insulin versus placebo or diet alone (Table 2). UGDP [10] and UKPDS 33 [11] were the only studies analysing mortality as a primary outcome. Eleven trials were graded as moderate quality, 9 as good. For all other studies, primary and secondary outcomes were surrogate endpoints whereas morbidity, mortality and hypoglycaemic events were reported as adverse events. Study characteristics are shown in Tables 1 and 2.

**Table 1:** Insulin versus hypoglycaemic drugs

Studies	Methodological quality: Low/moderate/good (Blinding Yes/No)	Participants (Ins/C)	TreatmentsTreatments	Follow-up (weeks)	Inclusion criteria	Primary outcome
Alvarsson 2009 [32]	Moderate (No)	51 (23/28)	Insulin/glibenclamide	104	FBG: [6; 12 mmol/L] 35–75 years	HbA1c, metabolic control
Aschner 2012 [33]	Good (No)	515 (250/265)	Met/S + Insulin Glargine/Sitagliptin	24	[7 %; 11] 35–70 years BMI = [25–45 kg/m^2]^	HbA1c
Bunck 2009 [34]	Moderate (No)	69 (33/36)	Met + Insulin glargine/exenatide	52	[6,5 %; 9,5] 35–75 years BMI = [25–40 kg/m^2]^	Metabolic control
Davies 2009 [35]	Good (No)	233 (117/118)	OHD (Met/S/TZD) + Insulin glargine/exenatide	51	[7,5 %;10 %] BMI > 27 kg/m^2^	HbA1c
Diamant 2010 [36]	Moderate (No)	467 (234/233)	OHD (Met/Met-S) + Insulin glargine/exenatide	26	[7 %; 11 %] - BMI : [25-40 kg/m^2^]	HbA1C
Gallwitz 2011 [37]	Moderate (No)	363 (181/182)	Met + Insulin Aspart/exenatide	26	[6,5 %; 10]	HbA1c
Gerstein 2006 [38]	Moderate (No)	405 (206/199)	OHD + Insulin glargine/conventional	24	[7,5 %; 11 %] BMI: [21–41 kg/m^2^] 18–80 years	HbA1c
Gerstein 2012 [39]	Good (No)	12527 (6273/6264)	Lifestyle recommendations and OHD + Glargine/Conventional care/omega3/placebo	6 years	Recent diabetes/Glucose Intolerance/	Composite of CV events
Hartemann 2011 [40]	Moderate (No)	27 (13/14)	OHD (Met/S) + Insulin NPH/Pioglitazone	24	[7,5 %; 9,5 %] BMI > 26 kg/m^2^ 18–80 years	HbA1c
Heine 2009 [41]	Moderate (No)	551 (267/282)	OHD (Met/S) + Insulin Glargine/Exenatide	26	[7 %; 10 %] BMI: [25–45 kg/m^2^] 35–75 years	HbA1c
Hollander 2009 [42]	Moderate (No)	217 (107/110)	OHD (Met + Sitagliptin) + Insulin Detemir/+/− S	26	[7,5 %; 10 %] BMI < 40 kg/m^2^	HbA1c
Nauck 2007 [43]	Moderate (No)	501 (248/253)	OHD (Met/S) + Insulin Aspart/Exenatide	52	[7 %; 11 %] BMI : [25–40 kg/m^2^] 30–75 years	HbA1c
Nauck 2012 [44]	Good (No)	1037 (364/351/322)	Met + S + Taspoglutide 10/taspoglutide 20/Insulin Glargine	24	[7 %; 10 %] BMI : [25–45 kg/m^2^] 18–75 ans	HbA1c
Reynolds 2007 [45]	Moderate (No)	40 (20/20)	OHD (Met/S) + Insulin Aspart/Rosiglitazone	26	[8 %; 12 %]	HbA1c, metabolic control
Rosenstock 2006 [46]	Good (No)	217 (105/112)	OHD (Met/S) + Insulin Glargine/Rosiglitazone	24	[7,5 %; 11 %] BMI > 25 kg/m^2^ >18 years	HbA1c
UKPDS 24 1998 [47]	Good (No)	458 (178/231/49)	Insulin/Chlorpropamide or Glyburide/Metformin	6 years	Diet failure UKPDS 33 25–60 years	HbA1c/FPG

**Table 2:** Insulin versus hypoglycaemic drugs and placebo or diet

Studies	Methodological quality: low, moderate, good (BlindingY/N)	Participants (I/C)	Treatment	Follow-up (weeks)	Inclusion criteria	Primary outcome
Blicklé 2009 [48]	Moderate (No)	102 (103/108)	OHD (Met/S) + Insulin Glargine/Lifestyle management	36 weeks	[7 %; 8 %] [40y; 75y] BMI: [24;35 kg/m^2^]	HbA1c
Russell Jones 2006 [49]	Good (No)	581 (234/115/232)	OHD (Met/S) + Insulin Glargine/Liraglutide placebo/Liraglutide	26	[7 %; 10 %] BMI < 40 kg/m^2^	HbA1c
UKPDS 33 [13]	Good (No)	3041 (911/1234/896)	Insulin/Chlorpropamide or glibenclamide/diet	10 years	25-65 years FBG 6,1–15 mmol/L	CV Morbimortality
UGDP [13, 50–55]	Good (Yes)	1027 (414/204/204/205)	Insulin/Tolbutamide/Phenformin/placebo	10 years	Diabete < 1 year	Mortality

*I* insulin, *C* control, *OHD* oral hypoglycaemic drugs, *Met* metformin, *S* sulphonylureas, *TZD* thazolidones, *BMI* body mass index, *CV* cardiovascular

### Primary outcomes

**Fig. 2:**
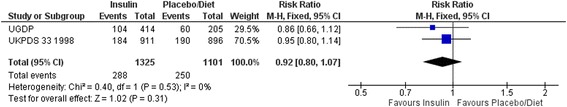
Death (Insulin vs. placebo/diet)

**Fig. 3:**
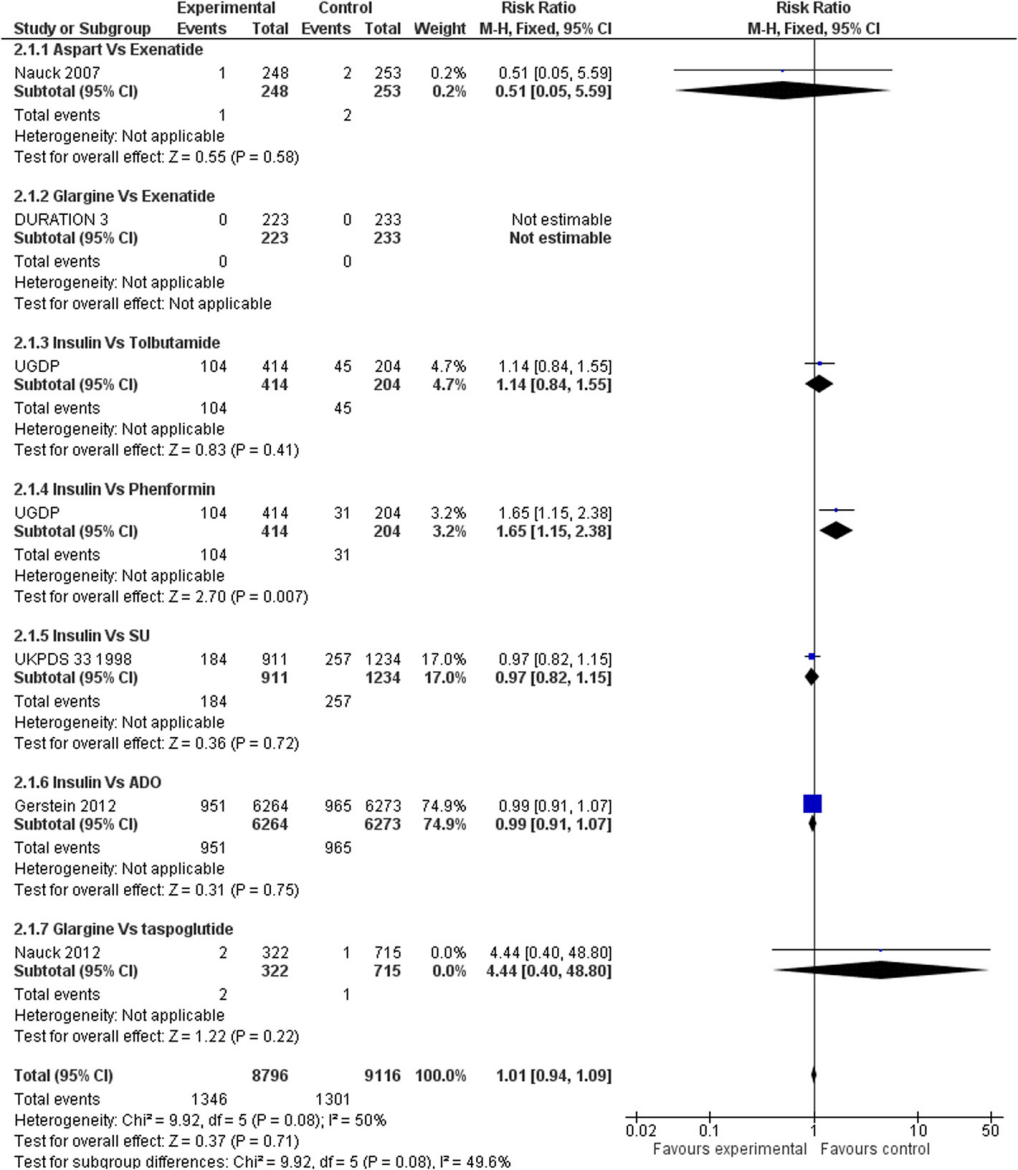
Death (Insulin vs. hypoglycaemic drug)

**Fig. 4:**
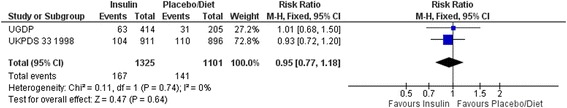
Cardiovascular death (Insulin vs. placebo/diet)

**Fig. 5:**
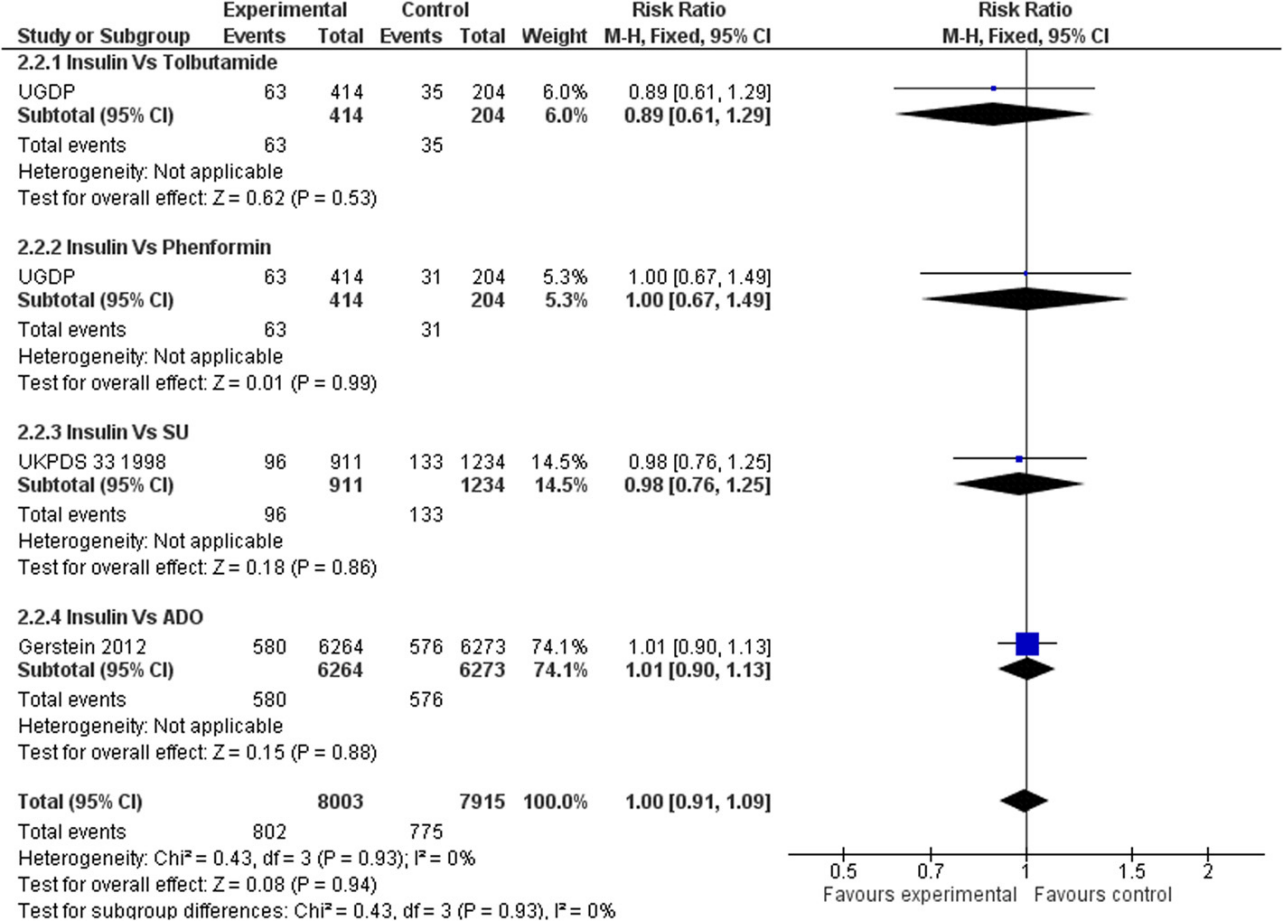
Cardiovascular death (Insulin vs. hypoglycaemic drug)

Insulin regimens did not affect mortality compared to placebo or diet alone (RR: 0.92, 95 % CI: 0.80 to 1.07) (Fig. 2). Results remained non-significant when insulin regimens were compared to hypoglycaemic drugs (RR: 0.99, 95 % CI 0.92–1.06) (Fig. 3). Insulin regimens showed no efficacy on cardiovascular mortality versus placebo/diet (RR: 0.95, 95 % CI 0.77 to 1.18) (Fig. 4), nor versus hypoglycaemic drugs (RR: 0.99, 95 % CI 0.90 to 1.09) (Fig. 5).

### Secondary outcomes

**Fig. 6:**
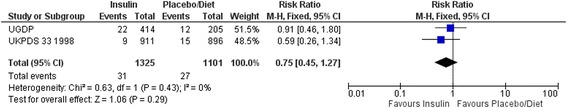
Sudden death (Insulin vs. placebo/diet)

**Fig. 7:**
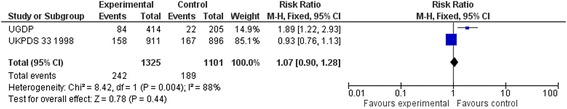
All myocardial infarctions (Insulin vs. placebo/diet)

**Fig. 8:**
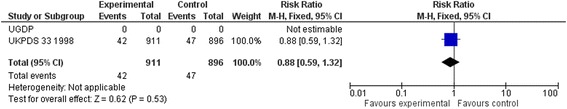
Stroke (Insulin vs. placebo/diet)

**Fig. 9:**
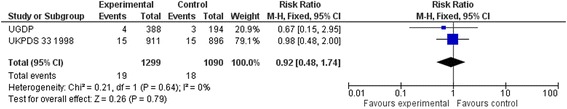
Amputation (Insulin vs. placebo/diet)

**Fig. 10:**
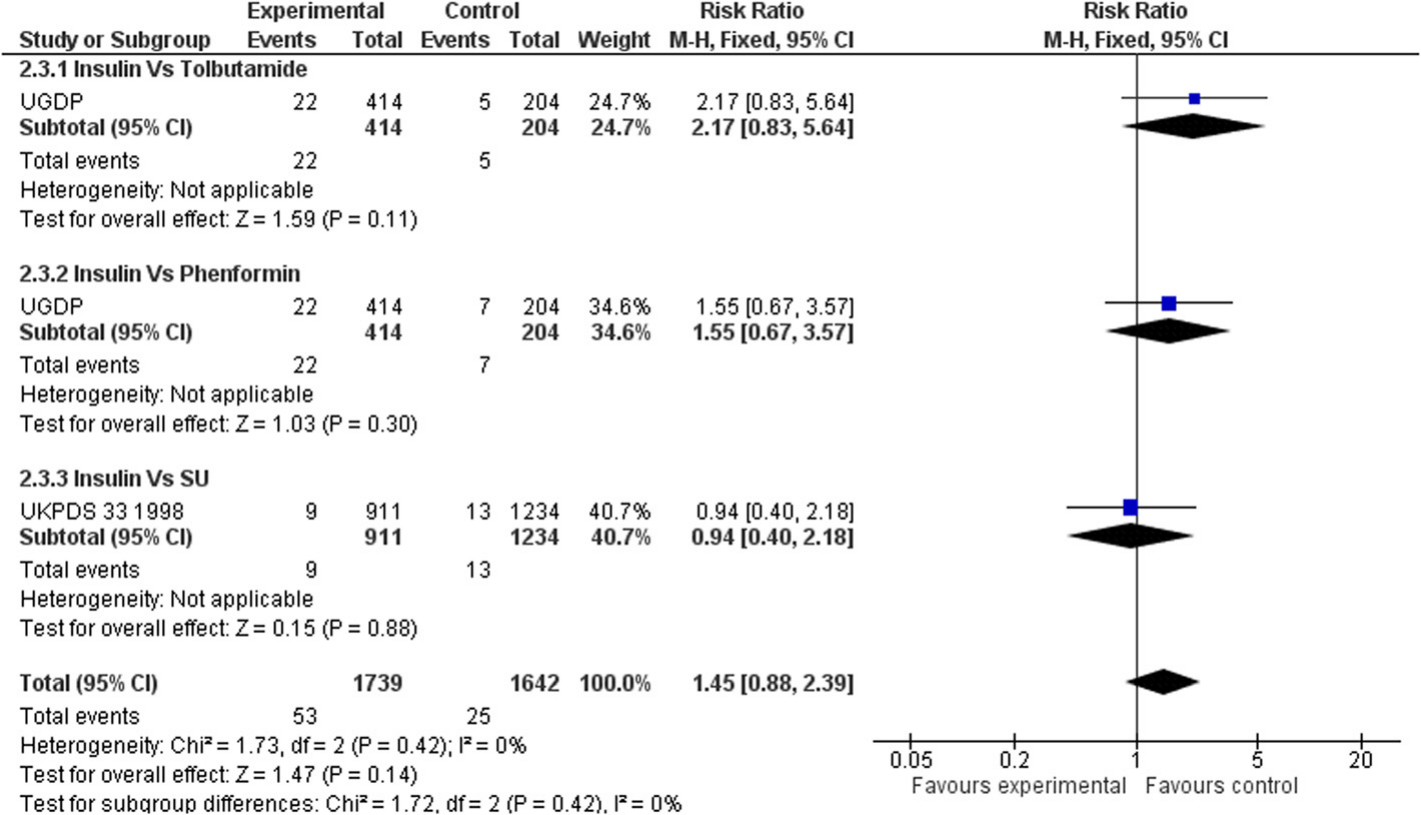
Sudden death (Insulin vs. hypoglycaemic drug)

**Fig. 11:**
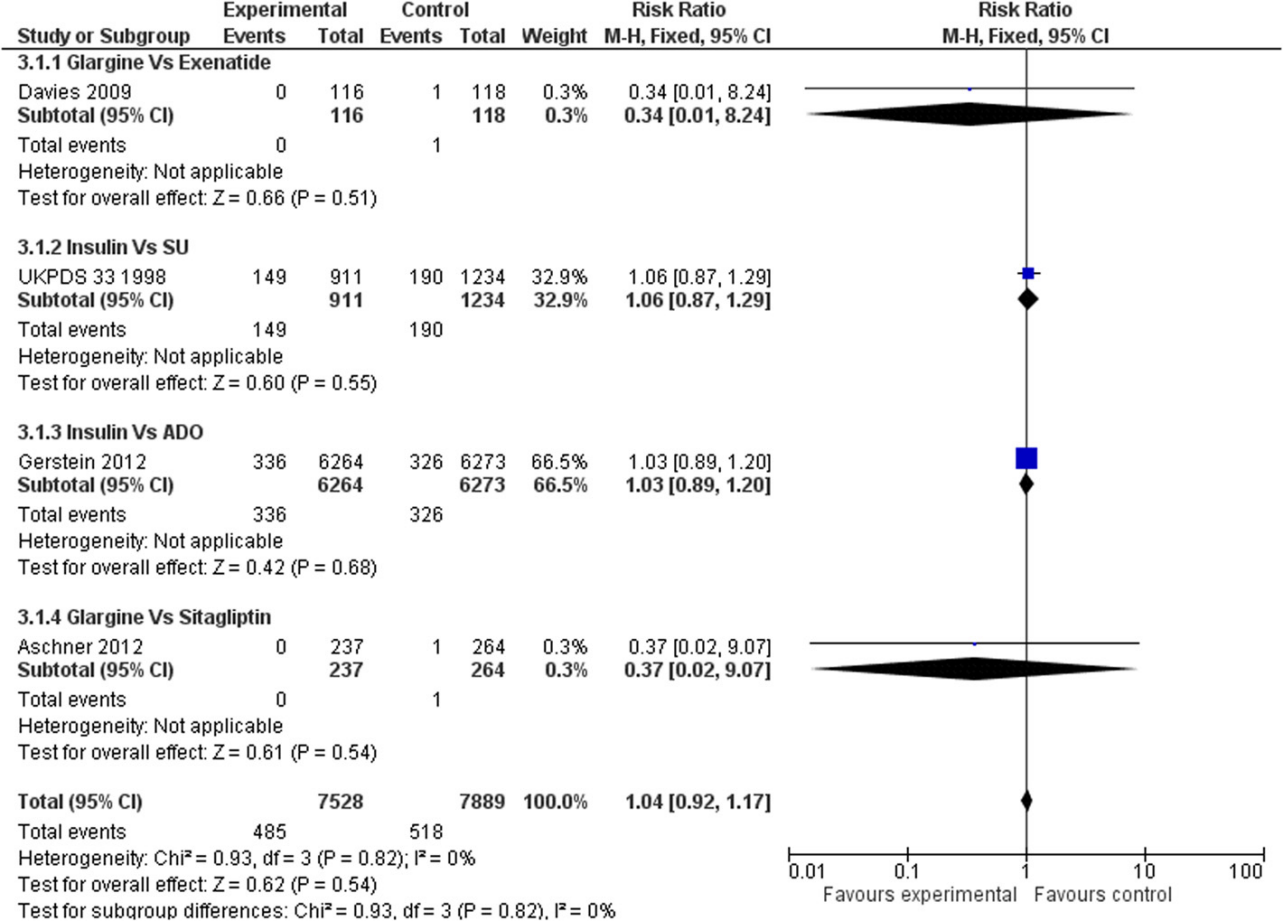
All myocardial infarctions (Insulin vs. hypoglycaemic drug)

**Fig. 12:**
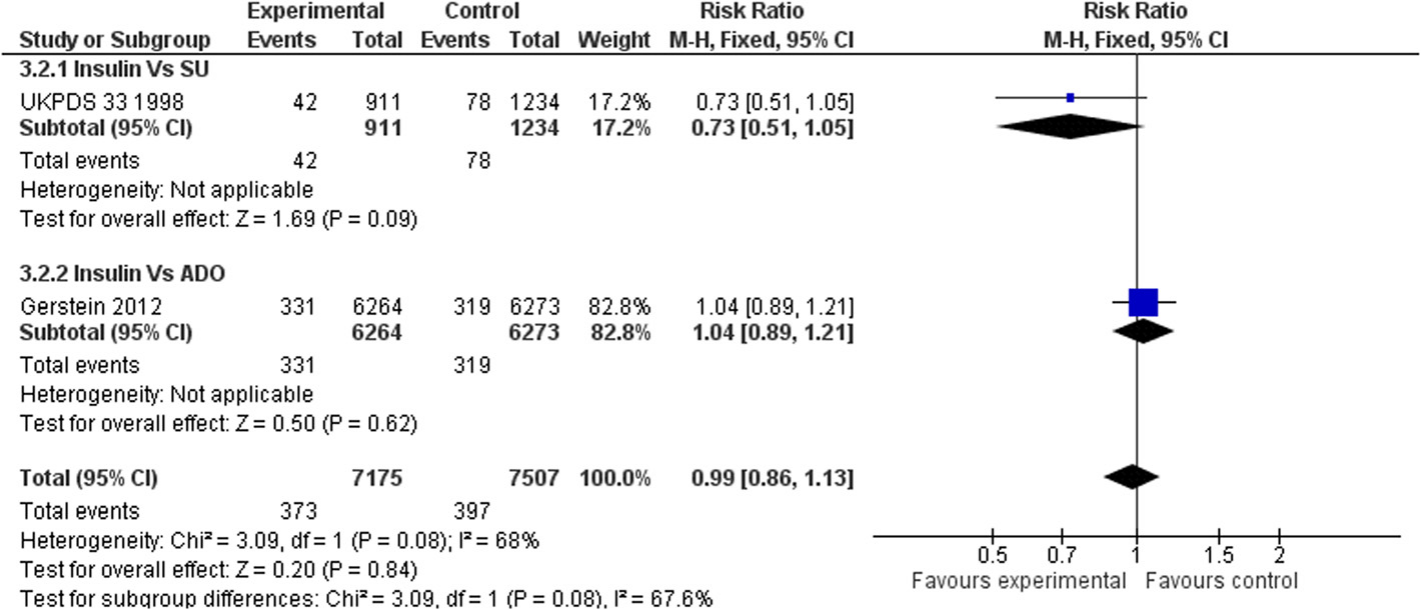
All strokes (Insulin vs. hypoglycaemic drug)

**Fig. 13:**
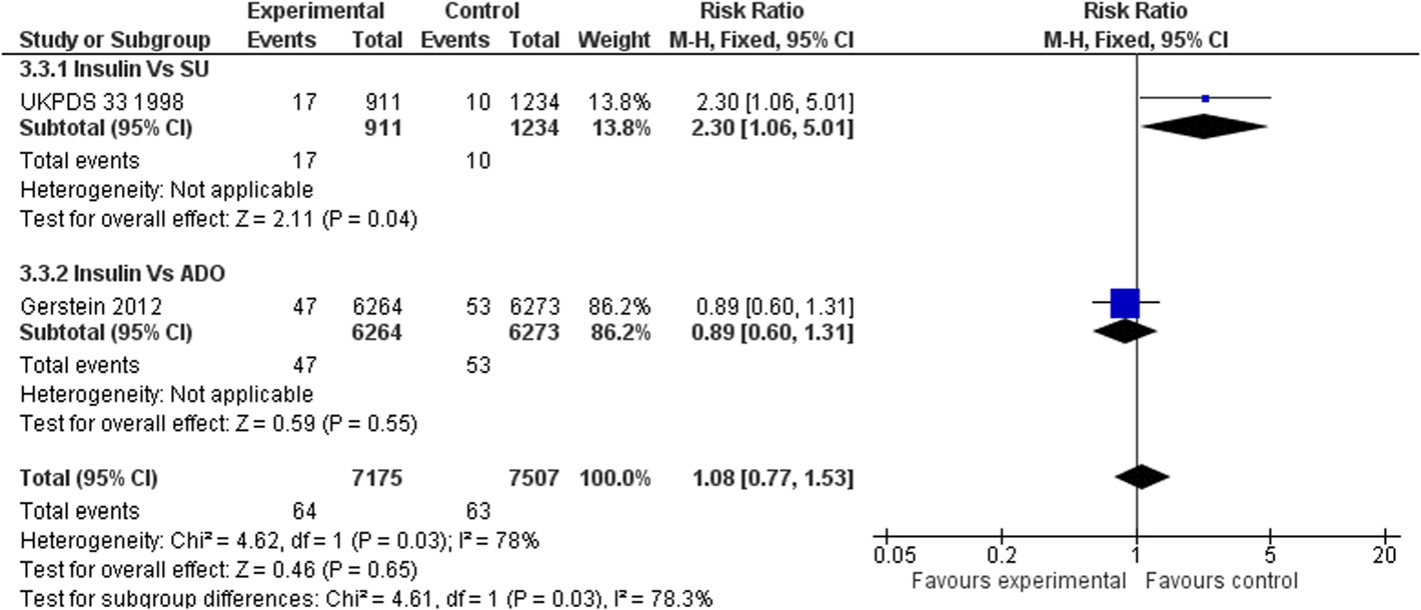
Amputation (Insulin vs. hypoglycaemic drug)

Compared to placebo or diet alone, insulin regimens did not affect sudden death (RR: 0.75, 95 % CI 0.45 to 1.27) (Fig. 6), myocardial infarction (RR: 1.07, 95 % CI 0.90 to 1.28) (Fig. 7), strokes (RR: 0.88, 95 % CI 0.59 to 1.32) (Fig. 8) or leg amputations (RR; 0.92, 95 % CI 0.48 to 1.74) (Fig. 9),

Results remained non-significant when insulin regimens were compared to hypoglycaemic drugs. (Figs. 10, 11, 12 and 13)

**Fig. 14:**
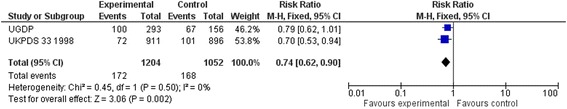
Blindness (Insulin vs. placebo/diet)

**Fig. 15:**
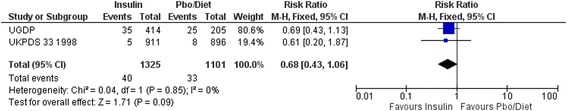
Renal Failure or doubling of serum creatinine (Insulin vs. placebo/diet)

Insulin regimens did not affect blindness (RR: 1.10, 95 % CI: 0.76 to 1.60) (Fig. 14), or renal failure or doubling of serum creatinine level (RR: 0.68, 95 % CI, 0.43 to 1.06) (Fig. 15), compared to placebo or diet alone.

Regarding retinal photocoagulation, the only available data was from UKPDS 33 [11] which compared insulin versus diet. In the insulin group, there was a significant decrease in retinal photocoagulations (RR: 0.70, 95 % CI: 0.53 to 0.94).

No data was available for neuropathy.

**Fig. 16:**
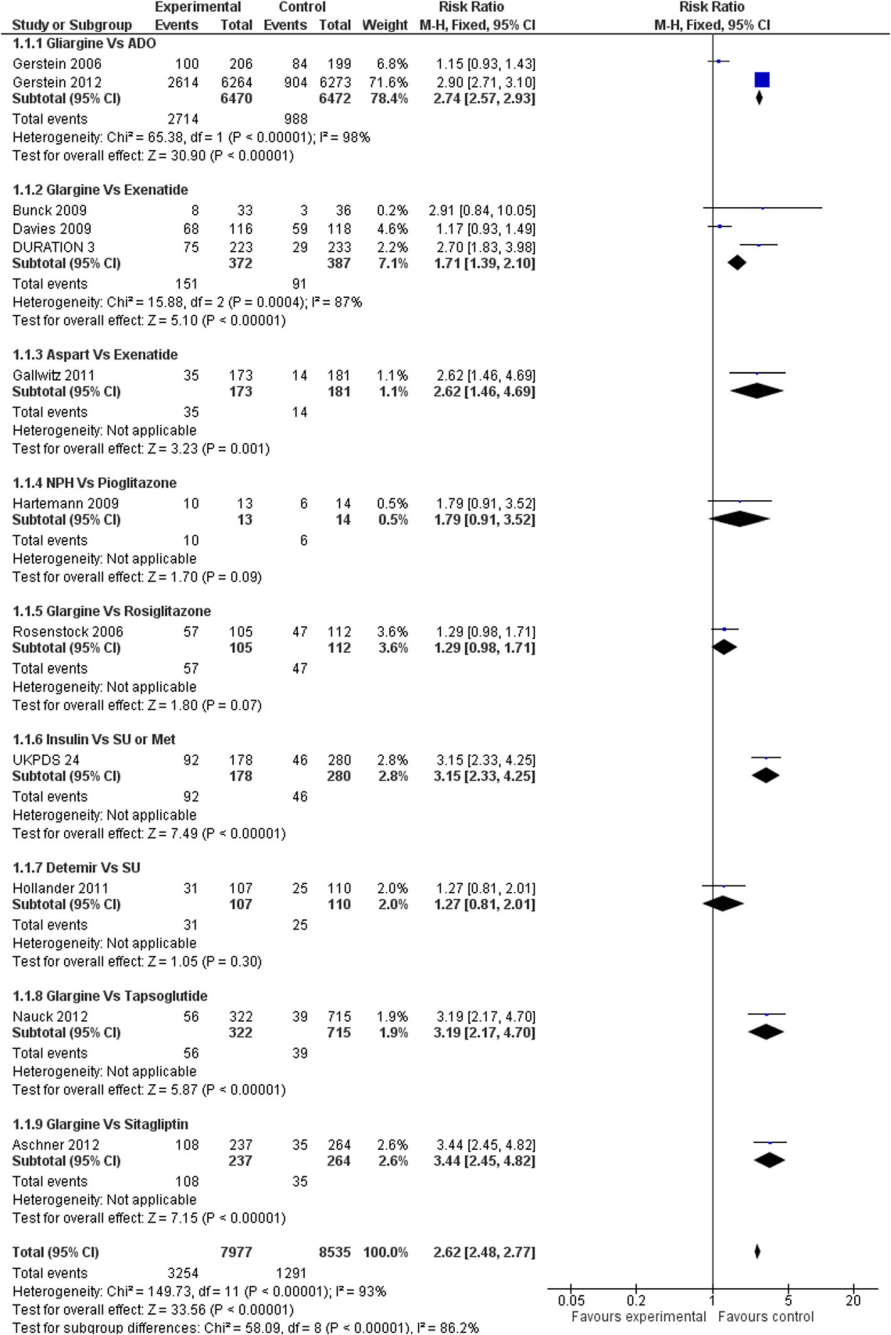
All hypoglycaemic events (Insulin vs. hypoglycaemic drug)

**Fig. 17:**
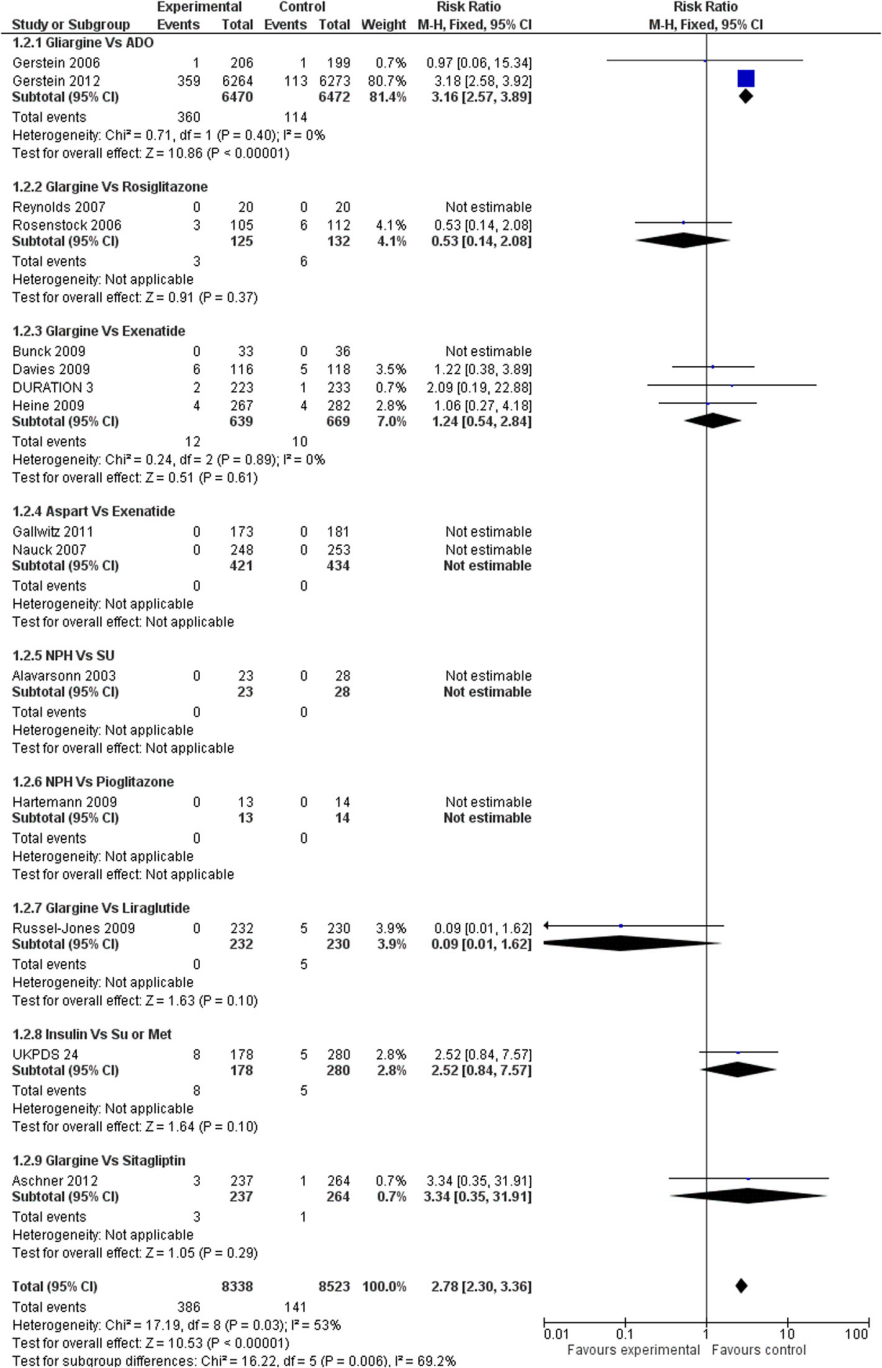
Major hypoglycaemic events (Insulin vs. hypoglycaemic drug)

Compared to oral hypoglycaemic drugs, the risk of hypoglycaemic events and major hypoglycaemia were significantly higher in the insulin group (RR: 2.62; 95 % CI 2.48 to 2.77 and RR: 2.78, 95 % CI 2.30 to 3.36 respectively) (Figs. 16 and 17)

Data from UGDP and UKPDS on these criteria have not been published and it was not possible to assess the effect of insulin vs. placebo/diet.

**Fig. 18:**
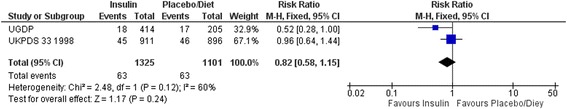
Death from cancer (Insulin vs. placebo/diet)

The only data available on death from cancer were from UKPDS [12] and UGDP [13], versus placebo/diet. Results were not significant, (RR: 0.82, 95 % CI 0.58 to 1.15) (Fig. 18). There is no data analysing insulin vs. hypoglycaemic drugs.

## Discussion

This meta-analysis from 20 RCT analysing 18,599 T2D patients showed no benefit of insulin vs. hypoglycaemic drugs or vs. diet/placebo on all-cause mortality, cardiovascular mortality, micro and macro vascular complications, except for retinopathy requiring photocoagulation. This last result comes from a single open label trial, and it is not possible to test its reproducibility. Moreover, regarding all types of hypoglycaemic events, insulin is significantly more harmful than other active treatments. There is no increase in death by cancer with insulin therapy vs. placebo/diet. (Fig. 18)

Data comparing the efficacy of insulin versus diet/placebo on cardiovascular mortality are scarce. Only two studies (2,426 patients) analysed mortality as a primary outcome: UGDP [10] and UKPDS 33 [11]. The other studies were based on surrogate outcomes, like HbA1c or other forms of ‘metabolic control’. Neither of these studies showed significant results regarding cardiovascular or all-cause mortality.

The small number of trials accessing these outcomes could explain the lack of significant results regarding all-cause mortality and cardiovascular mortality. Among these studies, two compare insulin with agents that have been withdrawn from the market, (tolbutamide, a sulfonylurea and phenformin, in the University Group Diabetes Program study). The sensitivity analysis removing these two studies did not affect the results, but cancelled heterogeneity across trials.

Results of our comparison with placebo/diet should be taken with caution, as we cannot exclude a 20 % reduction or a 7 % increase in all-cause mortality. We cannot exclude a reduction of 23 % or an 18 % increase in cardiovascular mortality either.

Clinical efficacy of insulin needs to be demonstrated with long-term trials. Insulin is currently prescribed to millions of patients without a proven benefit. The only two long-term studies available have significant weaknesses. The first, the UKPDS trial was open label and treatment could be increased according to blood glucose levels in the diet control group. 16 % of patients received insulin in the diet alone group [11]. Moreover, this study has major methodological shortcomings (was not double-blind, endpoints were added and follow-up was lengthened during the trial after observing negative results) [12–14]. Finally, concomitant treatments were not reported in the publications [15]. The second study was interrupted, because of increased mortality in the tested agent group, tolbutamide, which has been withdrawn from the market. The UGDP trial included T2D patients, according to diagnostic criteria in use at the time of patient recruitment (i.e., from 1961 to 1965) and was open label (comparisons: standard dose insulin vs adapted insulin vs placebo). After a 10-year follow-up (12.5 years on average), there were 15 to 18 % dropouts. The insulin regimens and concomitant treatments were different to those used today.

Our meta-analysis demonstrates no efficacy of insulin vs. placebo/diet on macrovascular complications (fatal or global). Furthermore, insulin does not decrease relevant microvascular outcomes such as blindness or renal failure either. However, significant results are found for retinopathy requiring laser photocoagulation. This effect should be taken with caution since it is derived from the single UKPDS trial, whose limits we have already underlined. The number needed to treat is about 30 at 10 years.

We analysed all types of insulin regimens, although “biphasic insulin aspart” or biphasic insulins are reported to induce a better metabolic control (estimated from fasting blood glucose and HbA1c) than other regimens (conventional insulin or other analogue insulin) [15]. We made this choice because there is no evidence that any insulin regimen was better at preventing clinically relevant outcomes like mortality or macro- or microvascular complications; and because there was too little data to analyse each single insulin regimen separately.

As the efficacy of insulin has not been proven, the safety analysis is essential. Our meta-analysis confirms that compared to other hypoglycaemic drugs, there is a serious risk of hypoglycaemia with insulin. According to UKPDS [11] the NNH is about 5 to 6 at 10 years for hypoglycaemia and 91 each year for a major hypoglycaemic event.

Previous meta-analyses have reported that various insulin regimens increase hypoglycaemia equally [16].

The link between severe hypoglycaemia and mortality has been reported in several studies [17] or, indirectly, when comparing conventional to intensive treatments [18–21]. Insulin treatment could therefore be considered as a high risk treatment option.

It is noteworthy that insulin is the second cause of drug-related hospital admissions in patients over 65 [22]. In two U.S. representative surveys over a 4 year period, Geller and al. estimated there were nearly 100,000 emergency department visits per year for insulin-related hypoglycaemia and errors [23], among which almost one-third required hospitalization. The estimated rate of severe neurological sequelae was 60 %. Patients over 80 treated with insulin were more than twice as likely to visit the emergency department and nearly 5 times as likely to be hospitalized. Moreover there is some evidence that hypoglycaemia may increase the risk of dementia [24].

Another well-known side effect of insulin-based regimen is weight gain [25], which secondarily increases insulin resistance.

The fact that insulin has shown no impact on clinically relevant outcomes is of major importance. Theoretically, insulin has potential negative clinical consequences, due to the underlying cellular and molecular mechanisms [3, 4]. From a patho-physiological point of view it is understandable that insulin is vital in the case of absolute insulin deficiency, such as type 1 diabetes. However, in T2D, where insulin resistance and high circulating levels of endogenous insulin are key concepts, the role of exogenous insulin is unclear. Observational studies have shown an association between endogenous insulin levels and cardiovascular risk, [26] and do not seem to be in favour of exogenous insulin either. In a retrospective cohort study, insulin therapy was associated with an increase in total mortality, (adjusted hazard ratio [HR] = 1.75; 95%CI: 1.24 to 2.47 for low insulin exposure and HR = 2.79; 95%CI: 2.36 to 3.30 for high insulin exposure, compared to no exposure) [27]. Other observational studies suggest an increased risk of cancer [28]. Our meta-analysis of RCTs shows no increase in deaths by cancer and cancer cases were not reported in many of the included studies. However, these results are to be taken with caution due to the possible lack of power of our meta-analysis. In vitro, the mitogenic effect of insulin is well established [29, 30].

The underlying mechanism of the higher risk associated with insulin is unclear. Positive associations could be explained by confounding factors: patients with T2D using insulin are usually older, with a longer history of diabetes, more comorbidities, at higher cardiovascular risk and with greater insulin resistance. Although observational studies may partially adjust for these factors, residual confounding factors may be responsible for the reported associations. However, it is also possible that hypoglycaemia plays a role (*via* sympathoadrenal activation, abnormal cardiac repolarization, increased thrombogenesis, inflammation and vasoconstriction [3, 4]) or that a direct atherogenic/mitogenic effect exists (cell growth, differentiation and proliferation [29, 30]), or that there is another specific effect of insulin that remains unknown.

### Implications for clinical practice

Insulin for T2D should only be used when no other treatment is available, to prevent short-term acute complications (such as hyperosmolar coma or ketoacidosis in case of an infection) or when the lack of insulin *per se* assigns patients in a high risk group.

This meta-analysis, as well as two other recent meta-analyses on metformin [7] and sulfonylureas [8], discredits blood glucose and HbA1c as valid surrogate outcomes for morbidity in T2D. The HbA1c target should be reconsidered since “the lower the better” model is censored by the increased mortality in the ACCORD study [18]. “The lower the better” and “treat to target” models, greatly increased requirements for insulin in patients with T2D (in the UK: 137,000 patients in 1991 vs. 421,000 in 2010 [31]).

The most appropriate treatment target in T2D is reduction in global cardiovascular risk. Although statins and angiotensin converting enzyme inhibitors have shown their efficacy to reduce cardiovascular mortality, for now, insulin has not.

### Implications for research

Further long-term studies are needed to establish whether insulin is beneficial in T2D.

## Conclusions

In T2D, insulin is recommended as an alternative or in combination with oral hypoglycaemic drugs when blood glucose targets are not achieved. Our meta-analysis does not support these recommendations, showing no long term benefit on cardiovascular risk or other clinical outcomes. Moreover our analysis has shown harmful adverse effects such as hypoglycaemia. The only benefit could be limited to reducing short term hyperglycaemia to improve symptoms (thirst, polyuria, asthenia, blurred sight) and to avoid acute complications (infection, hyperosmolar coma). Therefore, there is a great need for further studies.

## Abbreviations

95%CI, 95 % confidence interval; ADA/EASD, American Diabetes Association/European Association for the Study of Diabetes; HR, hazard ratio; NICE, National Institute for Health and Care Excellence; RR, risk ratio; T2D, type 2 diabetes; UGDP, University Group Diabetes Program; UKPDS, United Kingdom Prospective Diabetes study

## Additional files

Additional file 1: PRISMA checklist. (DOC 62 kb) Additional file 2: Appendix with search strategy. (DOCX 28 kb)
