# Supplementary material for: Efficacy and safety of insulin in type 2 diabetes: meta-analysis of randomised controlled trials
Source: BMC Endocr Disord. 2016 Jul 8;16:39. doi: 10.1186/s12902-016-0120-z (PMC4939045; doi:10.1186/s12902-016-0120-z)
Supplement: Additional file 2: — Appendix with search strategy. (DOCX 28 kb) [file 12902_2016_120_MOESM2_ESM.docx]

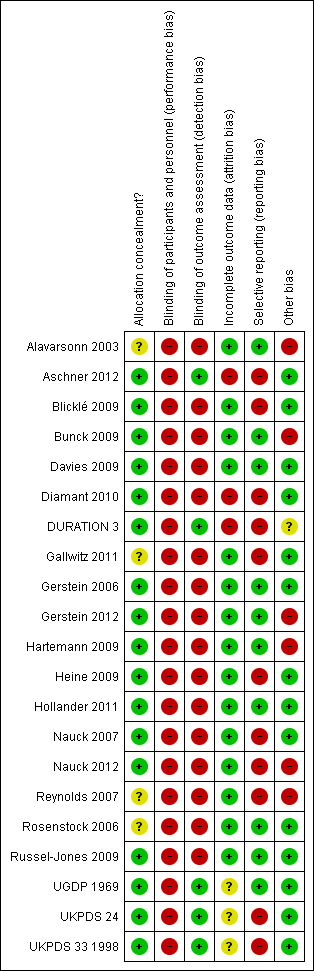


Risk of bias summary: review authors judgements about each risk of bias item for each included study

**Electronics Database**

**PUBMED**

((((((("Diabetes Mellitus, Type 2"[Mesh]) OR "Diabetes Mellitus"[Mesh])) NOT (((("Diabetes Insipidus"[Mesh] OR "Diabetes Insipidus, Neurogenic"[Mesh] OR "Diabetes Insipidus, Nephrogenic"[Mesh]) OR "Glucose Intolerance"[Mesh]) OR "Diabetes, Gestational"[Mesh]) OR "Diabetes Mellitus, Type 1"[Mesh]))) AND (("Hypoglycemic Agents"[Mesh]) OR ("Insulin"[Mesh] OR "Insulin, Lente"[Mesh] OR "Insulin Aspart"[Mesh] OR "Insulin Lispro"[Mesh] OR "Insulin, Short-Acting"[Mesh] OR "Insulin, Long-Acting"[Mesh])))) AND (((((((((((((((("Diabetes Complications"[Mesh]) OR "Mortality"[Mesh]) OR "Coronary Disease"[Mesh])) OR "Stroke"[Mesh]) OR "Peripheral Vascular Diseases"[Mesh]) OR "Diabetic Nephropathies"[Mesh]) OR "Diabetic Neuropathies"[Mesh]) OR "Diabetic Retinopathy"[Mesh]) OR "Kidney Diseases"[Mesh]) OR "Hypoglycemia"[Mesh]) OR ("Death, Sudden"[Mesh] OR "Death, Sudden, Cardiac"[Mesh]))) OR (microvascular disease)) OR (cardiovascular)) OR (macrovascular))

LIMITS : Randomized Controlled Trial /Controlled clinical Trial /Systematic reviews / Meta-analysis

from January 1^st^, 1950 to April 1^st^, 2013

**COCHRANE**

Diabetes Mellitus, Type 2 OR Diabetes Mellitus

NOT

Diabetes Insipidus OR Diabetes Insipidus, Neurogenic OR Diabetes Insipidus, Nephrogenic OR Glucose Intolerance OR Diabetes, Gestational OR Diabetes Mellitus, Type 1

AND

Hypoglycemic Agents OR Insulin OR Insulin, Lente OR Insulin Aspart OR Insulin Lispro OR Insulin, Short-Acting OR Insulin, Long-Acting OR Insulin Isophane OR insuline ultralente

AND

Diabetes Complications OR Mortality OR Coronary Disease OR Stroke OR Peripheral Vascular Diseases OR Diabetic Nephropathies OR Diabetic Neuropathies  OR Diabetic Retinopathy  OR Kidney Diseases  OR Hypoglycemia OR Death, Sudden OR Death, Sudden, Cardiac OR microvascular disease  OR cardiovascular OR macrovascular

AND

Randomized Controlled Trial OR Controlled clinical Trial OR Systematic reviews OR Meta-analysis

**EMBASE**

1.Diabetes Mellitus, Type 2"[Mesh])  2. Diabetes Mellitus"[Mesh]))
3. 1 OR 2
4. Diabetes Insipidus"[Mesh] 5. Diabetes Insipidus, Neurogenic 6. Diabetes Insipidus, Nephrogenic[Mesh]

7. Glucose Intolerance"[Mesh] 8. Diabetes, Gestational"[Mesh] 9. Diabetes Mellitus, Type 1"[Mesh])))

10. OR (4-9)
11.3 NOT 10
12.Hypoglycemic Agents"[Mesh]  13.Insulin"[Mesh] 14.Insulin, Lente"[Mesh] 15.Insulin Aspart"[Mesh]
16.Insulin Lispro"[Mesh] 17.Insulin, Short-Acting"[Mesh] 18.Insulin, Long-Acting"[Mesh]
19. Insulin Isophane 20. insuline ultralente
21. OR (12-20)
22.11 and 21

23.Diabetes Complications"[Mesh]  24.Mortality"[Mesh] 25.Coronary Disease"[Mesh]

26.Stroke"[Mesh] 27.Peripheral Vascular Diseases"[Mesh]  28.Diabetic Nephropathies"[Mesh]

29.Diabetic Neuropathies"[Mesh]  30.Diabetic Retinopathy"[Mesh]  31.Kidney Diseases"[Mesh]

32.Hypoglycemia"[Mesh] 33.Death, Sudden"[Mesh] 34.Death, Sudden, Cardiac"[Mesh]

35.microvascular disease  36.cardiovascular disease 37.macrovascular
38.OR (23-37)
39.22 and 38
40.Randomized Controlled Trial/  41.Controlled clinical Trial/ 42.Systematic reviews/ 43.Meta-analysis
44.OR (40-43)
45.39 and 44
46.limit 45 to human
47.limit 46 to exclude medline journals
